# Tailoring nonlinear optical properties of Bi_2_Se_3_ through ion irradiation

**DOI:** 10.1038/srep21799

**Published:** 2016-02-18

**Authors:** Yang Tan, Zhinan Guo, Zhen Shang, Fang Liu, Roman Böttger, Shengqiang Zhou, Jundong Shao, Xuefeng Yu, Han Zhang, Feng Chen

**Affiliations:** 1 School of Physics, State Key Laboratory of Crystal Materials and Key Laboratory of Particle Physics and Particle Irradiation (Ministry of Education) Shandong University Shandong, Jinan, 250100, China; 2 Helmholtz-Zentrum Dresden-Rossendorf, Institute of Ion Beam and Materials Research, Bautzner Landstrasse 400, 01328 Dresden, Germany; 3 SZU-NUS Collaborative Innovation Center for Optoelectronic Science and Technology, Key Laboratory of Optoelectronic Devices and Systems of Ministry of Education and Guangdong Province, College of Optoelectronic Engineering, Shenzhen University, Shenzhen 518060, P.R. China; 4 Institute of Biomedicine and Biotechnology, Shenzhen Institutes of Advanced Technology, Chinese Academy of Sciences, Shenzhen 518055, P.R. China

## Abstract

The nonlinear optical property of topological insulator bismuth selenide (Bi_2_Se_3_) is found to be well-tailored through ion irradiation by intentionally introducing defects. The increase of the optical modulation depth sensitively depends on the careful selection of the irradiation condition. By implementing the ion irradiated Bi_2_Se_3_ film as an optical saturable absorber device for the Q-switched wave-guide laser, an enhanced laser performance has been obtained including narrower pulse duration and higher peak power. Our work provides a new approach of tailoring the nonlinear optical properties of materials through ion irradiation, a well-developed chip-technology, which could find wider applicability to other layered two-dimensional materials beyond topological insulators, such as graphene, MoS_2_, black phosphours etc.

Two-dimensional (2D) materials have received great scientific and technical attentions in research areas of physics, chemistry, and materials sciences^1^,^2^. In optics, peculiar optical performances have been found in 2D materials including polarization dependent absorption and nonlinear optical absorption^3^,^4^,^5^. Besides, 2D materials can be easily integrated with photonic structures such as waveguides and cavities, since the surfaces of 2D materials can be passivated without any dangling bonds. Based on the unique optical properties of 2D materials, many important optical applications have been realized in integrated photonics, including waveguide polarizers, mode-locked, waveguide amplifier^6^ and Q-switched waveguide lasers^3^,^7^,^8^,^9^,^10^,^11^. Taking the Q-switched pulsed waveguide laser for example, a waveguide structure with doping of rare-earth ions could be used as the gain medium and 2D materials with the saturable absorption property can be coated onto the waveguide facet as broadband optical saturable absorbers.

Properties of 2D materials can be tailored by doping and external electric field. Recently, it has been demonstrated that the ion irradiation can also induce beneficial effects to nanostructured materials^12^,^13^,^14^,^15^. The interaction of energetic particles (e.g. electrons or ions) normally generates atomic defects in the target and changes properties of the materials. In spite of defects, the ion irradiation process may bring a positive effect to the material, in some cases. For example, the atomic constitution and morphology of graphene can be changed in a controllable manner by ion irradiation^16^. For nanotubes, ion irradiation can interconnect and merge the nanotubes, induce extreme pressure and lead to the formation of fullerene like “onions” in the nanotube^17^. In addition, it has been proved that the mechanical, electronic and even magnetic properties of nanostructured materials can be tailored by ion irradiation^18^,^19^,^20^. Nevertheless, there have been no reports on tailoring the optical properties of 2D materials by ion irradiation as of yet.

The Bi_2_Se_3_ film, as a prototype topological insulator (TI), possesses an excellent saturable absorption and has been applied in Q-switched laser as the saturable absorber^21^,^22^. In this work, we applied the ion irradiation to modify the nonlinear optical property of the Bi_2_Se_3_ film in constructive way. The Bi_2_Se_3_ films have been irradiated with nitrogen ions to diverse, precisely controlled fluences. It has been found that the saturable absorption of the Bi_2_Se_3_ film was enhanced after irradiation. The modification of the nonlinear optical absorption has shown a direct relation to the irradiation-induced defects in Bi_2_Se_3_. Through a careful control of the defect concentration, the saturable absorption of Bi_2_Se_3_ was intentionally tailored. The irradiated Bi_2_Se_3_ film was integrated with a neodymium doped yttrium aluminum garnet (Nd:YAG) waveguide for the passively Q-switched waveguide laser generation. Based on the modulation of the irradiated Bi_2_Se_3_ film, the Q-switched pulses with shorter pulse duration and a higher peak power were obtained, demonstrating the advantages of the irradiation treatment on the optical properties of 2D materials.

## Results and Discussion

### Bi_2_Se_3_ nanoplatelets

**Figure 1:**
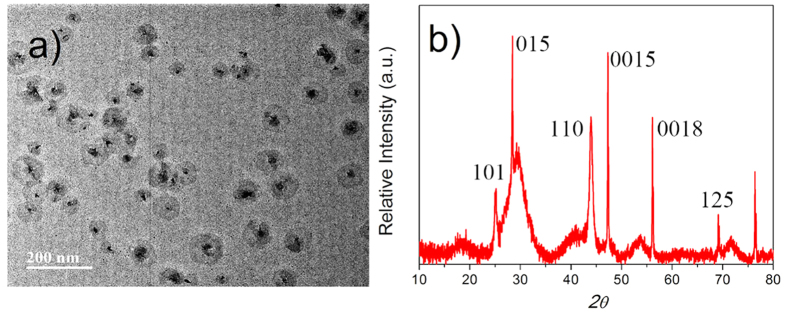
Bi_2_Se_3_ nanoplatelets. SEM image (**a**) and the XRD diffraction pattern (**b**) of Bi_2_Se_3_ NPs.

The Bi_2_Se_3_ nanoplatelets (NPs) used in the current study were synthesized via a polyol method. During the Bi_2_Se_3_ NPs synthesis, multiple reagents were added into a 25.0 mL two-neck flask with a teflon-coated magnetic stirring bar, including 0.10 g of bismuth (III) nitrate pentahydrate (Bi(NO_3_)_3_⋅5H_2_O), 0.05 g of sodium selenite (Na_2_SeO_3_), 0.22 g of polyvinyl pyrrolidone (PVP), and 10.0 mL of ethylene glycol (EG). The flask was connected to a reflux condenser and heated by a heating mantle. After heating the solution to 190 ^o^C for 2 hours under constant stirring, the flask was removed from the heating mantle and the Bi_2_Se_3_ NPs were chemically grown in the solution, the SEM image is shown in Fig. 1a. The XRD pattern of the Bi_2_Se_3_ NP is displayed in Fig. 1b indicating the good crystallinity of the as-produced NPs. Then, the solution was cooled, centrifuged, washed (with isopropyl alcohol), and dropped onto a silica wafer. Dried at 60 °C, the solution was volatilized and Bi_2_Se_3_ NPs overlapped with each other constituting a 2D material film on the silica wafer. The thickness of the Bi_2_Se_3_ film was measured to be 180 nm (±5 nm) by the atomic force microscope (AFM).

### Optical properties of irradiated Bi_2_Se_3_ films

**Figure 2:**
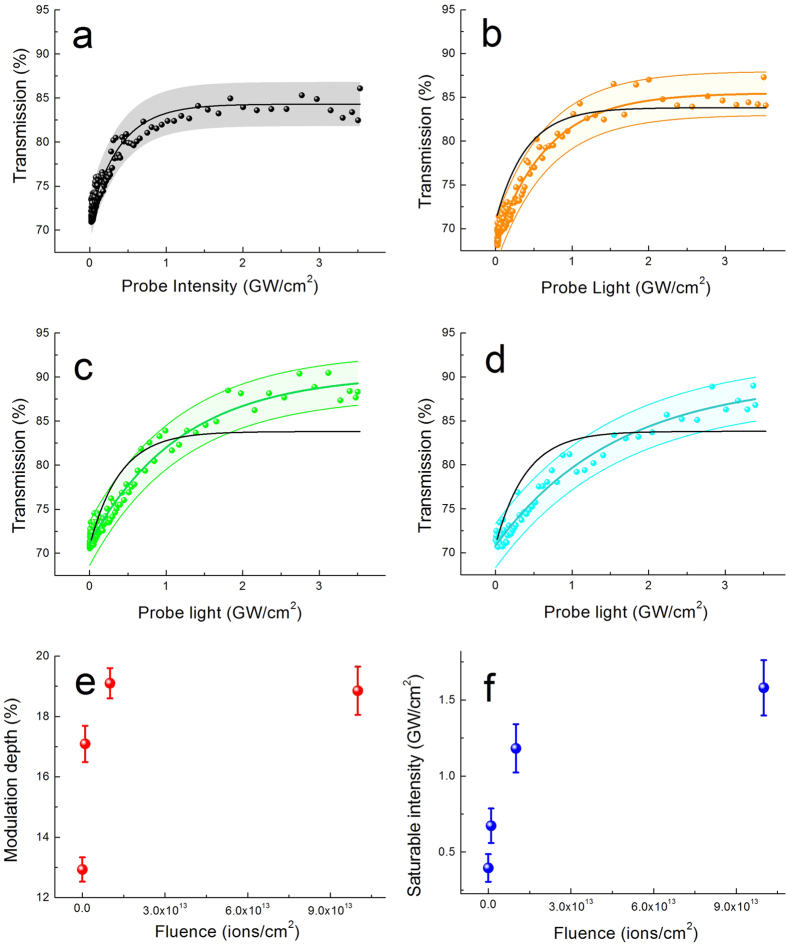
Optical properties of irradiated Bi_2_Se_3_ films. Nonlinear transmission as a function of the probe light intensity of S_0_ (**a**), S_1_ (b), S_2_ (**c**) and S_3_ (**d**). Color solid lines are fitted curves. Black lines in (**a**–**c**) are fitted curves of S_0_ for comparison. Color bands are error bands (±2.5%). The modulation depth (**e**) and the saturable intensity (**f**) of the irradiated Bi_2_Se_3_ film under different fluence.

Three pieces of Bi_2_Se_3_ films were irradiated by N^+^ ions at a series of fluences, which were labeled as S_1_ (1 × 10^12 ^ions/cm^2^), S_2_ (1 × 10^13 ^ions/cm^2^) and S_3_ (1 × 10^14 ^ions/cm^2^), respectively. One piece of the Bi_2_Se_3_ film without irradiation was used as the control/reference sample (S_0_). The measured nonlinear transmission of Bi_2_Se_3_ films is shown in Fig. 2. As we can see, the clear saturable absorption of the Bi_2_Se_3_ films was observed in all three samples. However, there are remarkable differences in the nonlinear transmission induced by ion irradiation in S_1_, S_2_ and S_3_. The modulation depth (Δ*T*) and the saturable intensity (*I*_sat_) of the Bi_2_Se_3_ films are increased with the irradiation fluence.

To quantitatively determine the saturable absorption property of Bi_2_Se_3_ film, the relation between the transmission (*T*) and the excitation energy (*I*) has been fitted by the following formula^23^:


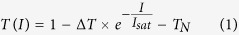
where *T*_N_ is the nonsaturable absorbance. The fitted values of the optical parameters are shown in Fig. 2e,f. Δ*T* and *I*_sat_ of the original sample (S_0_) are 12.9% and 0.4 GW, respectively. The value of *I*_sat_ can be tailored by the fluence of N^+^ ions. In the case of low-fluence irradiation (less than 1 × 10^13 ^ions/cm^2^), *I*_sat_ has a rapid change. By further increasing the ion fluence, the variation is smaller and tends to saturation. For the modulation depth, there is a sensitive response of the Bi_2_Se_3_ film to the irradiation treatment at the low-fluence regime. And the value of Δ*T* is adjusted from 12.9% to 19% by the fluence. With a fluence of more than 1 × 10^13^ ions/cm^2^, Δ*T* reaches a saturation value of 19%.

Based on the previous discussion, the saturable absorption of the Bi_2_Se_3_ film can be tailored by ion irradiation. Utilizing the irradiated Bi_2_Se_3_ film as the saturable absorber, the performance of the Q-switched pulse laser is expected to be improved. For example, the pulse duration of the Q-switched pulse laser can be expressed by the equation below^24^,^25^:


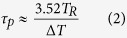
where *τ*_p_ is the pulse duration and *T*_R_ is the cavity round-trip time. A shorter pulse duration can be obtained by increasing the modulation depth. Besides, the saturable intensity of the Bi_2_Se_3_ film is increased after irradiation. Therefore, a higher energy of the excitation laser is required in order to reach the saturation of the irradiated Bi_2_Se_3_ film, suggesting that the pulse energy of the Q-switched pulse laser can be further increased.

### Mechanism of tailoring the nonlinear optical properties

**Figure 3:**
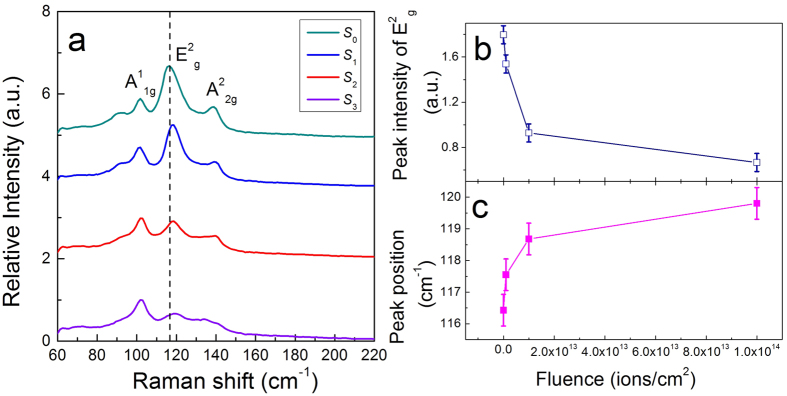
Raman spectra of irradiated Bi_2_Se_3_ film. (**a**) Raman spectra of S_0_, S_1_, S_2_, and S_3_. The peak intensity (**b**) and peak position (**c**) variation of E^2^_g_ with the fluence.

In order to explain the mechanism of tailored nonlinear optical properties, the Raman spectra of Bi_2_Se_3_ films were measured by a confocal microscope. Figure 3a shows the measured Raman spectra and the variation of the *E*^2^_g_ peak with respect to different irradiation fluences. As one can see, phonon peaks have blue shifts and their intensity is decreased. The change of the phonon peak (*E*^2^_g_) directly relates to the ion fluence. The variation of the Raman spectra indicates the deformation or damage of the Bi_2_Se_3_ structure by the ion irradiation.

**Figure 4:**
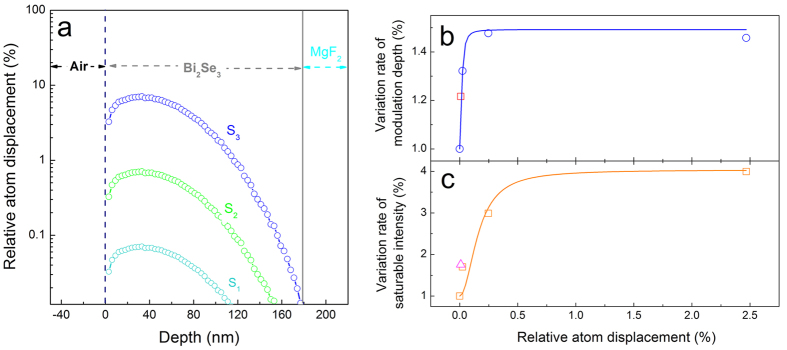
Mechanism of tailoring the nonlinear optical properties. (**a**) The relative atom displacement of the irradiated Bi_2_Se_3_ films along with the depth. (**b**,**c**) are the variation rate of the modulation depth (circles) and the saturable intensity (squares) with different relative atom displacement.

During the irradiation process, the incident energetic ions lost their energy, which was transferred from nitrogen ions to the host atoms in the Bi_2_Se_3_ film and caused the displacement of atoms in the Bi_2_Se_3_ (defects/damage of the structure) through both inelastic and elastic collisions with the host atoms (also known as electronic and nuclear energy losses). The amount of structural damage in the Bi_2_Se_3_ film can be calculated by the Monte Carlo simulation SRIM (Stopping and Range of Ions in Matter)^26^. To simplify the discussion, the irradiation-induced structure damage is numerically represented by the relative atom displacement (RAD) in the Bi_2_Se_3_ film (percentage of the disordered atoms in the Bi_2_Se_3_ film). Figure 4a shows the distribution of RAD along with the depth from the sample surface towards to the bulk. The damage induced by irradiation is different along the depth and is concentrated near the surface (depth less than 80 nm). Besides, the damage is nearly proportional to the ion fluence of irradiation. Increasing the ion fluence from 1 × 10^12^ to 1 × 10^14 ^ions/cm^2^, the average RAD of the Bi_2_Se_3_ is increased by two orders of magnitude, that is, 0.025% (*S*_1_), 0.247% (*S*_2_) and 2.467% (*S*_3_), respectively.

The relationship between the average RAD of the Bi_2_Se_3_ and the nonlinear optical parameters is displayed in Fig. 4b,c. With the damage less than 0.25%, Δ*T* and *I*_sat_ have a rapid change and the amplitudes of the variation are 1.4% and 3.2%, respectively. At high-damage level (more than 0.25%), the value of parameters tends to saturate.

According to the relationship between the RAD and the nonlinear optical property (the saturable absorption), the optical performance of the Bi_2_Se_3_ can be precisely controlled by irradiation. The interaction between the incident ions and the Bi_2_Se_3_ film can be simulated by SRIM. And the calculated RAD can give us a rough conjecture of the nonlinear optical properties of the irradiated Bi_2_Se_3_.

## Discussion

Ion irradiation has been demonstrated as a novel method to tailor the nonlinear optical property of the Bi_2_Se_3_ film. Through the control of the ion fluence, the saturable absorption properties can be modulated efficiently. This work suggests that ion irradiation is an efficient way to modify the nonlinear optical property of 2D materials, which suggests broad applications in ultrafast laser photonics. Utilizing the ion-irradiated Bi_2_Se_3_ films as the optical saturable absorbers, the performance of the Q-switched waveguide laser is supposed to be enhanced.

**Figure 5:**
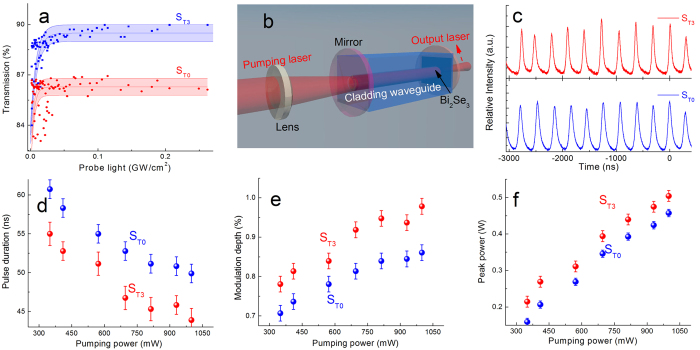
Application of the irradiated Bi_2_Se_3_ film. (**a**) Nonlinear transmission of S_T0_ (red circles) and S_T3_ (blue circles). (**b**) Experimental setup for the Q-switched pulse waveguide laser. The variation of the pulse duration (**d**), modulation depth (**e**) and peak power (**f**) as a function of the pumping power, modulated by S_T0_ (red circles) and S_T3_ (blue circles), respectively.

In this work, the irradiated Bi_2_Se_3_ film was utilized as the passive saturable absorber for the Q-switched waveguide laser emission. In order to reduce the saturable intensity of the film, S_3_ was thinned by the mechanical exfoliation. The material near the surface of S_3_ was removed and the remaining Bi_2_Se_3_ has a thickness of 10 nm (labeled as S_T3_). According to the simulation result shown in Fig. 4a, the average RAD in *S*_T3_ was 0.01%. S_0_ was also thinned to the 10 nm by the same technology as the control sample (labeled as *S*_T0_). The measured nonlinear transmissions of S_T0_ and S_T3_ are shown in Fig. 5a. The saturable intensity and the modulation depth of S_T0_ are found to be 4.7 MW/cm^2^ and 5%, respectively. In S_T3_, the values of *I*_sat_ and *T* are increased by a factor of 1.76 and 1.22, respectively. The variation rates of optical parameters in S_T3_ are pointed in Fig. 4b,c (the red square and the pink triangle, respectively). As one can see, the measured values of S_T3_ show a good agreement with previous results.

The experimental setup for the Q-switched waveguide laser is shown in Fig. 5b. S_T1_ and S_T0_ were compressed onto the output facet of a Nd:YAG waveguide as the saturable absorber, respectively. With the pumping power above 300 mW at 810 nm, a stable pulse laser emission is observed under the modulation of S_T0_ and S_T3_, respectively. The pulse trains of the output laser are shown in Fig. 5c under a pumping power of 350 mW.

The pulse duration and the peak power of the output laser are shown in Fig. 5d,e, respectively. According to the measured pulse duration, the modulation depth can be calculated by Equation (2) and is displayed in Fig. 5e. The pulse laser modulated by S_T3_ has a higher peak power and larger modulation depth than S_T0_ under the same pumping condition. The measured parameters of the output pulse laser demonstrate the better performance of the irradiated Bi_2_Se_3_ as the saturable absorber, such as a higher peak power and a shorter pulse duration.

## Methods

### Ion irradiation

Four pieces of Bi_2_Se_3_ films were prepared by the same technology for this work. Three of them were irradiated by N^+^ ions with an energy of 30 keV at different fluences, labeled as *S*_1_ (fluence of 1 × 10^12 ^ions/cm^2^), *S*_2_ (fluence of 1 × 10^13 ^ions/cm^2^) and *S*_3_ (fluence of 1 × 10^14 ^ions/cm^2^), respectively.

### Nonlinear transmission measurement

The nonlinear absorption coefficient of the Bi_2_Se_3_ film was measured by the Z-scan technology. A 1064-nm laser with 22-ps pulse duration and an energy of 0.5 μJ was focused using a lens (a focal distance of 400 mm), resulting in ∼24.5 μm beam waist. For an accurate measurement, a large-aperture lens was used for collecting the transmitted laser light. By moving the Bi_2_Se_3_ film to the focal point, the power of the transmitted light was measured as a function of the energy density of the probe light.

### Mechanical exfoliation of the Bi_2_Se_3_ film

The Bi_2_Se_3_ was mechanically exfoliated by the ordinary adhesive tape: Lightly pasting the tape onto the surface of the Bi_2_Se_3_ film and then uncovering the adhesive tape. The Bi_2_Se_3_ will stick to the surface of the tape and be stripped. After repeating this operation, the thickness of the Bi_2_Se_3_ will be reduced to the desired thickness.

### Q-switched waveguide laser

Figure 4b shows the schematic for the generation of Q-switched waveguide lasers. The waveguide structure was fabricated by the femtosecond laser writing^27^. The double-cladding waveguide was inscribed into a neodymium doped yttrium aluminum garnet (Nd:YAG) ceramic sample (doped by 2% Nd^3+^ ions, offered by Baikowski Ltd.). The diameters of the inner and outer tubular cladding are 30 μm and 100 μm, respectively. The detailed information of the waveguide fabrication was reported in ref. 28.

The Nd:YAG ceramic waveguide was used as the gain medium for the laser emission at the wavelength of 1064 nm. A mirror with a reflectivity of 99.98% was coated onto the input facet of the waveguide as the input mirror. While, a Bi_2_Se_3_ film was compressed tightly onto the output facet. The resonant cavity was composed by the Nd:YAG ceramic waveguide, the input mirror and the Bi_2_Se_3_ film. A continuous-wave optical pump at the wavelength of 810 nm was utilized in this work from a tunable Ti:Sapphire laser (Coherent MBR PE). The pump beam was focused by a convex lens (focal length 25 mm) and coupled into the Nd:YAG ceramic waveguide passing through the input mirror. As the Q-factor of this resonant cavity was modulated by the saturable absorption of the Bi_2_Se_3_ film on the output facet, the emission of the pulsed waveguide laser can be obtained.

## Additional Information

**How to cite this article**: Tan, Y. *et al*. Tailoring nonlinear optical properties of Bi_2_Se_3_ through ion irradiation. *Sci. Rep.* **6**, 21799; doi: 10.1038/srep21799 (2016).
