# Author Correction: Tailoring nonlinear optical properties of Bi_2_Se_3_ through ion irradiation

**DOI:** 10.1038/s41598-021-97241-3

**Published:** 2021-08-30

**Authors:** Yang Tan, Zhinan Guo, Zhen Shang, Fang Liu, Roman Böttger, Shengqiang Zhou, Jundong Shao, Xuefeng Yu, Han Zhang, Feng Chen

**Affiliations:** 1 School of Physics, State Key Laboratory of Crystal Materials and Key Laboratory of Particle Physics and Particle Irradiation (Ministry of Education) Shandong University Shandong, Jinan, 250100 China; 2 Helmholtz-Zentrum Dresden-Rossendorf, Institute of Ion Beam and Materials Research, Bautzner Landstrasse 400, 01328 Dresden, Germany; 3 SZU-NUS Collaborative Innovation Center for Optoelectronic Science and Technology, Key Laboratory of Optoelectronic Devices and Systems of Ministry of Education and Guangdong Province, College of Optoelectronic Engineering, Shenzhen University, Shenzhen, 518060 P.R. China; 4 Institute of Biomedicine and Biotechnology, Shenzhen Institutes of Advanced Technology, Chinese Academy of Sciences, Shenzhen, 518055 P.R. China

Correction to: *Scientific Reports* 10.1038/srep21799, published online 18 February 2016

The original version of this Article contains a repeated error, where “TEM” images are incorrectly referred to as “SEM” images.

In the Results and Discussion section, subheading “Bi_2_Se_3_ nanoplatelets”.

“After heating the solution to 190 °C for 2 hours under constant stirring, the flask was removed from the heating mantle and the Bi_2_Se_3_ NPs were chemically grown in the solution, the SEM image is shown in Fig. 1a.”

should read:

“After heating the solution to 190 °C for 2 hours under constant stirring, the flask was removed from the heating mantle and the Bi_2_Se_3_ NPs were chemically grown in the solution, the TEM image is shown in Fig. 1a.”

In the legend of Fig. 1,

“SEM image (**a**) and the XRD diffraction pattern (**b**) of Bi_2_Se_3_ NPs.”

should read:

“TEM image (**a**) and the XRD diffraction pattern (**b**) of Bi_2_Se_3_ NPs.”

